# Aspects épidémiologiques, cliniques, cytologiques et immunophénotypiques des leucémies aiguës chez les enfants: expérience du laboratoire d'hématologie du Centre Hospitalier Universitaire IBN Sina

**DOI:** 10.11604/pamj.2016.23.258.8396

**Published:** 2016-04-28

**Authors:** Mariam Doumbia, Jean Uwingabiye, Aboubacar Bissan, Razine Rachid, Souad Benkirane, Azlarab Masrar

**Affiliations:** 1 Laboratoire d'Hématologie, Centre Hospitalier Universitaire IBN SINA, Faculté de Médecine et de Pharmacie de Rabat, Université Mohammed V, Maroc

**Keywords:** Leucémie aiguë, enfant, épidémiologie, cytologie, immunophénotypage, Acute leukemia, child, epidemiology, cytology, immunophenotyping

## Abstract

L'objectif de ce travail était de décrire les caractéristiques épidémiologiques, cytologiques et immunophénotypiques des leucémies aigues (LA) chez les enfants diagnostiqués au Centre Hospitalo-universitaire (CHU) Ibn Sina et de déterminer aussi la concordance entre les résultats de la cytologie à ceux de l'immunophénotypage. Il s'agit d'une étude transversale réalisée au laboratoire d'hématologie du CHU Ibn Sina entre Juin 2012 et Mai 2014. Parmi 104 cas de LA diagnostiqués, 52% étaient des garçons avec un sex-ratio H/F= 1,32 et l’âge médian de 5,7 ans. La répartition des différents types de LA était: LA lymphoïde (LAL) (74%), LA myéloïde(LAM) (20,2%), LA biphénotypique(LAB) (65,8%). Parmi les LAL,78% ont été classé LAL B et 22% comme LALT. Les signes cliniques étaient principalement présentés par le syndrome tumoral (73,1%), la fièvre (61%) et syndrome hémorragique (50%). Les anomalies de l'hémogrammeles plus fréquents étaient: thrombopénie (89,4%), anémie (86,5%), hyperleucocytose (79,8%). Le taux des blastes périphérique et médullaires était statistiquement élevé pour LAL que pour LAM et LAB (p<0,001). Le taux de rechute et de mortalité était respectivement de 21,2% et16, 3%. Le taux de concordance entre les résultats de la cytologie et ceux de l'immunophénotypage était de 92,7% pour LAL et de 82,6% pour LAM. Le diagnostic des LA se base toujours en premier sur la cytologie. L'immunophénotypage nous a permis de faire une meilleure distinction entre les leucémies aiguës. La prise en charge des LA pédiatriques est un problème majeur qui nécessite les centres spécialisés.

## Abstract (en)

The aim of this study was to describe epidemiological, cytologic and immunophenotypic aspects of acute leukemias (AL) in children diagnosed at IBN SINA University Hospital Center and to determine the concordance between cytology and immunophenotyping results. This is a cross-sectional study conducted in the hematology laboratory of IBN SINA University Hospital Center between June 2012 and May 2014. Among the 104 cases with diagnosed AL, 52% were boys with a sex-ratio H/F= 1.32, the average age was 5.7 years. The distribution of different types of AL was: lymphoid AL (LAL) (74%), myeloid (AML) (20.2%), biphenotypic AL (BAL) (65.8%). Among the LALs, 78% were classified as B LAL and 22% as T LAL. Clinical signs were mainly presented with tumor syndrome (73.1%), fever (61%) and hemorrhagic syndrome (50%). The most common blood count abnormalities were: thrombopenia (89.4%), anemia (86.5%), hyperleukocytosis (79.8%). The rate of peripheral and bone marrow blasts was statistically higher for LAL than for AML and BAL (p <0.001). The rate of relapse and mortality was 21.2% and 16. 3% respectively. Concordance rate between the results of cytology and of immunophenotyping was 92.7% for LAL and 82.6% for AML. Diagnosis of AL is always based primarily on cytology. Immunophenotyping allowed us to make a better distinction between acute leukemias. The management of paediatric AL is a major health problem which requires specialized care centers.

## Introduction

Les leucémies aiguës (LA) constituent un groupe hétérogène d'hémopathies malignes caractérisées par la prolifération clonale et incontrôlée de précurseurs hématopoïétiques bloqués dans leur différenciation [1]. Ils représentent entre 10 et 15% des hémopathies malignes avec un taux d'incidence standardisé à la population mondiale inférieur à 6/100 000 habitants/an [2] et un âge de survenue qui varie selon le type de leucémie [1]. Chez l'enfant, les leucémies aiguës myéloïdes (LAM) sont rares et surviennent avant l’âge de 2 ans ou après 15 ans. À l'inverse, La leucémie aiguë lymphoblastique (LAL) est le cancer le plus fréquent chez les enfants et représente environ un quart de tous les cancers chez les sujets de moins de 15 ans [3] et elle est aussi environ 5 fois plus prédominent que la LAM [4]. Le diagnostic des LA repose avant tout sur des critères cytologiques et immunophénotypiques des blastes de la moelle osseuse [1, 4]. L'apport de l'immunophénotypage puis de la cytogénétique et enfin de la biologie moléculaire ont permis de décrire de plus en plus d'entités [1, 4]. Etant donné que ces méthodes spécifiques sont couteuses et non disponibles dans les centres hospitaliers des pays en voie de développement, la cytologie garde sa place dans le diagnostic de LA [5]. L'objectif de ce travail était de décrire les caractéristiques épidémiologiques, cytologiques et immunophénotypiques des LA chez les enfants diagnostiqués au Centre Hospitalo-universitaire (CHU) Ibn Sina de Rabat et de déterminer aussi la concordance entre les résultats de la cytologie à ceux de l'immunophénotypage.

## Méthodes

Il s'agit d'une étude transversale réalisée au laboratoire d'hématologie du CHU Ibn Sina de Rabat du 1^er^ Juin 2012 jusqu'au 30 Mai 2014 portant sur les enfants chez lesquels une LA a été diagnostiquée. Tous les données concernant les aspects épidémiologiques, cliniques, biologiques, immunophénotypiques et évolutifs des leucémies aigues ont été recueillis à partir des dossiers des enfants admis au service d'hémato-oncologie pédiatrique duCHU IBN SINA. Ont été exclu les patients qui n'ont pas bénéficié l'immunophénotypage pour confirmation de la cytologie et ceux dont les dossiers étaient incomplets. L'hémogramme a été réalisé à l'aide de l'automate «Dx 5000» à partir des échantillons de sang prélevés par ponction veineuse sur des tubes EDTA (acide éthylène diamine tétracétique). La ponction de la moelle osseuse a été pratiquée au niveau de l’épine iliaque postérieure. Les frottis de sang et de moelle ont été colorés au *MGG (May-Grünwald-Giemsa)* par la méthode automatique (DMO). Pour chaque patient, trois lectures indépendantes des frottis de sang et de moelle ont été assurées et validées par des cytologistes. Le diagnostic de LA a été porté dès qu'il y avait plus de 20% de blastes dans la moelle osseuse selon les critères de la classification de l'organisation Mondiale de la santé (OMS) 2008 [1, 4]. La recherche de l'activité myéloperoxydasique par la technique à la pyronine a été appliquée sur un frottis de moelle dans le cas où l'aspect myéloïde n’était pas évident et elle a été considérée négative si on notait une proportion de moins de 3% de blastes peroxydase positive. L'immunophénotypage a été réalisée à l'aide d'un cymomètre en flux FC 500(Beckman)qui possède un large panel d'anticorps monoclonaux appartenant aux différentes lignées hématopoïétiques: lignée lymphoïde T (anti-CD2, CD3, CD3c, CD4, CD5, CD7, CD8, CD56), lignée lymphoïde B (anti-CD19, CD20, CD22, CD22c, CD79a),la lignée myéloïde (anti MPO, CD11c, CD13, CD14, CD15, CD33, CD36, CD65, CD117), et la lignée érythroïde (anti-glycophorine A) et les marqueurs des cellules souches( anti-HLA Dr, CD10, CD34). Le seuil de positivité était fixé à 20% pour les marqueurs membranaires et pour les marqueurs cytoplasmiques il a été conclu positif lorsqu'il était présent sur au moins 10% des cellules étudiées. Les données ont été analysées à l'aide d'un logiciel de traitement statistique SPSS version 10 au département d’épidémiologie de la Faculté de Médecine et de Pharmacie de Rabat. Les résultats ont été exprimés sous forme de médiane et intervalle interquartiles (IQ) pour les variables quantitatives, sous forme d'effectif et de pourcentages pour les variables qualitatives. Le test de Kruskall-Wallis a été utilisé pour comparer plus de deux variables quantitatives ayant une distribution asymétrique. Pour comparer deux variables qualitatives, l'analyse statistique s'est appuyée sur le test de Khi deux ou de Fischer exact. Dans tous les cas, une valeur p < 0,05 a été retenue comme seuil de signification.

## Résultats

Le Tableau 1 montre les caractéristiques épidémiologiques, cliniques, hématologiques et évolution clinique des patients en fonction de type de leucémie. Pendant la période d’étude, 104 cas de LA ont été diagnostiqués: LAL (77/104=74%), LAM (21/104=20,2%), leucémie aigue biphénotypique(LAB) (6/104=5,8%). Parmi les LAL,78% (60cas) ont été classé comme LAL B et 22% (19 cas) comme LALT. Parmi les enfants présentant une LA: 54 étaient des garçons (52%) et 50(48%) étaient des filles avec un sex-ratio H/F de 1,32. L’âge médian des patients était de 5,7 ans avec IQ de [3-9 ans]. L’âge médian des patients atteint de LAM (9ans, IQ: [5-12]) étaient statistiquement élevé par rapport à celui des patients atteint de LAL (5ans, IQ: [2-8ans]) et de LAB (5,3 ans, IQ: [3,1-8,5]) (p=0,02). Les signes cliniques ont été enregistrés chez 90,4% des patients (94 cas). Le syndrome tumoral a été retrouvé dans 73,1% des cas, fièvre dans 61% et syndrome hémorragique dans 50%. L'association de ces 3 signes a été retrouvée dans 22,1%. La différence des signes cliniques entre les différents types de leucémie n’était pas statistiquement significative sauf pour la fièvre qui était plus fréquente chez les patients atteints de LAL (70,1%) que chez ceux atteints de LAB (57,1%) et LAL (30%) (p=0,004). Tous les patients ont présenté au moins une anomalie de l'hémogramme; une thrombopénie a été observée dans 89,4% des cas, une anémie dans 86,5%, une hyperleucocytose dans 79,8% des cas, une leucopénie dans 10,6% des cas et une pancytopénie dans 4,8% cas. Le taux médian de blastes circulants était 88%, IQ: 74-92% et la répartition de taux de blastes périphériques en fonction de type de leucémie était: LAL (médiane: 83,2%; IQ: 48-95%), LAB (médiane 76%; IQ: 55-89%) et LAM (médiane 50%; IQ: 19-81%) (p<0,001). L’étude du myélogramme a objectivé un taux médian de blastes de 90%, IQ: 79-93%. La différence des taux des blastes médullaires entre les différents types de leucémies était statistiquement significative (p<0,001) avec un taux médian de blastes de: 90,4%; IQ: 87-99% dans le LAL; 81%; IQ: 77-89% dans le LAB et 78%; IQ: 53-88% dans le LAM. Dans les LAM; les signes de disgranulopoïèse, de disérythropoïèse et de dismégacaryopoïèse ont été retrouvé respectivement chez 45%,37% et 13% des patients. Le taux de rechute pour l′ensemble des patients était de 21,2%(22 cas) et Il était significativement élevé chez les patients atteints de LAM (52,5%) que chez ceux atteints de LAL (27,3%) et LAB (14,3%) (p=0,02). Le taux de mortalité était de 16,3%. Les pourcentages des cas positifs pour chaque marqueur sont représentés dans laFigure 1 pour les LAL et par la Figure 2 pour les LAM. Le Tableau 2 résume la confrontation des résultats de la cytologie avec ceux de la cytométrie en flux. Le taux de concordance entre les résultats de la cytologie et ceux de l'immunophénotypage était de 92,7% pour les LAL et de 82,6% pour les LAM.

**Figure 1:**
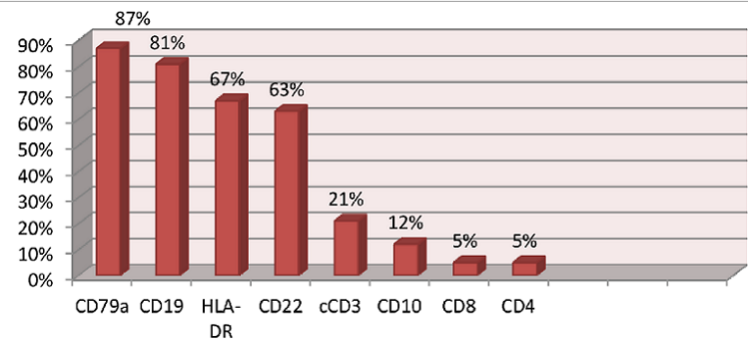
Le pourcentage des CD positifs pour chaque marqueurs lymphoïdes dans les LAL

**Figure 2:**
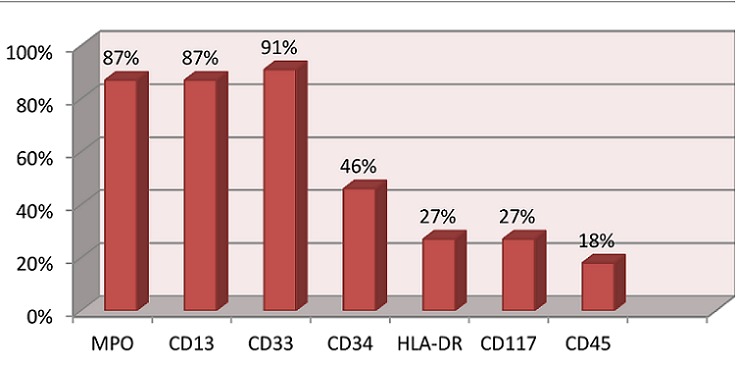
Le pourcentage des CD positifs pour chaque marqueur myéloïde dans les LAM

**Tableau 1:** Caractéristiques épidémiologiques, cliniques, hématologiques et évolution clinique des patients en fonction de type de leucémie

Paramètres	Total N=104	LAL N=77	LAM N=21	LAB N=6	*p*
**Données socio-démographiques**					
Sexe* M	54 (51,9)	44(57,1)	8(38,1)	2(33,3)	0,186
F	50(48,1)	33 (42,9)	13(61,9)	4(66,7)	
Sex-ratio M/F	1,12	1,33	0,61	0,5	
Age**	5,7[3 - 9]	5 [2,8 - 8]	9[5 -12]	5,3[3,1-8,5]	0,02
**Hémogramme**					
Leucocytes (G/L) **	24,9[7,9- 62,2]	25 [7,9-78,4]	13,6 [7,5-44,9]	19,7 [9,7-81,4]	0,68
Hémoglobines (G/L) **	6 [4,5-8,3]	6 [4,7-8,8]	5,8 [4-7,5]	7[5-8]	0,36
Plaquettes (G/L) **	35 [12-78]	44 [13-79,5]	19 [7,5-60]	15[12-57]	0,32
**Anomalies de l'hémogramme**					
Anémie*	90(86,5)	65(84,4)	20(95,2)	5(99)	0,42
Thrombopénie*	93(89,4)	68(88,3)	19(90,5)	6(100)	0,659
Hyperleucocytose	83(79,80)	67(87,01)	16(84,21)	6(75)	0,13
Leucopénie*	11(10,6)	7(9,1)	2(9,5)	1(10)	0,17
Pancytopénie*	5(4,8)	4(5,19)	1(5,26)	0(0)	0,15
**Frottis sanguin**					
Blastes(%) **	88 [74 - 92]	83,2 [48-95]	50 [19-81]	76[55-89]	<0,001
**Myélogramme**					
Blastes(%) **	90 [79 - 93]	90,4 [87-99]	78 [53-88]	81[77-89]	<0,001
**Signes cliniques**					
Syndrome tumoral*	76(73,1)	57(74)	13(65)	6(85,7)	0,53
Fièvre*	64(61,5)	54(70,1)	6(30)	4(57,1)	0,004
Syndrome					
hémorragique*	52(50)	37(48,1)	8(40)	4(57,1)	0,58
**Evolution clinique**					
Rechute après traitement*	22(21,2)	21(27,3)	12(52,2)	1(14,3)	0,02
Décès*	17(16,3)	10(13)	6(30)	1(14,3)	0,18

* Les données ont été représentées sous forme de N(%)

** Les données ont été représentées sous forme de médiane [Intervalle interquartile], LAL: Leucémie aigue lymphoblastique, LAM: Leucémie aigüe myéloïde, LAB: Leucémie aigue biphénotypique

**Tableau 2:** Confrontation entre la cytologie et l'immunophénotypage

CYTOLOGIE	IMMUNOPHENOTYPAGE	% de CONCORDANCE
LAL 83 cas	LAL: 77 cas	92,7%
	LAB: 6	
LAM 21 cas	LAM: 19 cas	82,6%
	LAB: 2 cas	

LAL: Leucémie aigue lymphoblastique, LAM: Leucémie aigüe myéloïde, LAB: Leucémie aigue biphénotypique

## Discussion

La leucémie est la plus fréquent des cancers pédiatriques et il existe deux principaux types de LA; LAL et LAM [6]. Dans notre étude, LAL était plus fréquent (74%) suivi par LAM (20,2%) et LAB (5,8%), et LAL B représentait 78% des LAL. Nos résultats confirment ceux d'autres auteurs qui ont montré que la LAL représente près de 80% de la leucémie chez les enfants âgés de 0-14 ans [7] avec une incidence annuelle allant jusqu′à 40 cas par million dans les pays d'Europe occidentale et jusqu′à 30-35 cas par million dans les pays d′Europe orientale, mais moins de 20 par million en Afrique subsaharienne [8]. La LAL B représente plus de 80% de l′ensemble de LAL [9, 10]. Selon les données de la littérature, les LAL de type T ont un très mauvais pronostic chez les enfants et les LAL de type B mature ont un bon pronostic [11]. Le deuxième type le plus fréquent de la leucémie aigue chez l′enfant est LAM avec une incidence stable de 5-9 cas par million par an dans le monde entier [7, 8]. L’âge est le facteur de pronostic le plus important pour la réussite du traitement d'induction des LAM [12]. L’âge médian de la survenue de LA était de 5,7 ans avec une différence statistiquement significative entre les patients atteint de LAM et ceux atteint de LAL (9ans vs 5ans). Ceci peut être expliqué par la prédominance de LAL chez les enfants contrairement à la LAM [13]. Nos résultats montrent la prédominance des LAL chez le sexe masculin. L'augmentation du rapport H/F est corrélée à une augmentation de la rechute de type (rechute testiculaire) chez les patients atteints de LAL. Contrairement aux LAL, le sexe ne semble pas être un facteur de pronostic dans les LAM [2]. Les leucémies aiguës surtout chez les enfants s'associent toujours à des degrés variables des signes de prolifération et d'insuffisance médullaire. Les enfants atteints de LA présentent les symptômes suivants: fatigue, fièvre, infection persistante, ecchymoses ou saignement, douleur osseuse, arthralgies ou adénopathies [14, 15]. Dans notre étude, la présentation clinique de la LAM était la même que la LAL sauf la fièvre qui était significativement plus fréquente chez les patients atteints de LAL (70,1%) que chez ceux atteints de LAB (57,1%) et LAL (30%). Les anomalies de l'hémogramme dans notre étude étaient principalement représentées par la thrombopénie (89,4%), l'anémie (86,5%) et l'hyperleucocytose (79%). Ces résultats sont similaires à ceux de la littérature [5, 16]. L'intensité de la thrombopénie et le risque hémorragique sont des signes fréquents dans les leucémies [16]. L'hyperleucocytose constitue un facteur pronostique majeur; le pronostic est plus favorable quand la leucocytose est inférieure à 100 000/mm^3^ [5]. Contrairement à une hyperleucocytose, une leucopénie qui est une chose inhabituelle dans les LA surtout lymphoblastique a été retrouvée chez 10% des patients dans cette étude. Nos données montrent aussi qu'il y avait une différence statistiquement significative de taux des blastes entre les différents types de leucémies au niveau du sang périphérique et de la moelle osseuse.

Dans notre étude, le taux de rechute était significativement élevé chez les patients atteint de LAM (52,2%) que ceux atteint de LAL (27,3%) et LAB (14%) et le taux de mortalité était de 16,3%. Le traitement de la LAL pédiatrique représente un succès de la médecine moderne avec 85 à 90% de survie à l'heure actuelle, alors que dans les années 1970, le taux de survie était d’à peine 15% [16]. L'amélioration de la survie en pédiatrie a été rendue possible en partie grâce à des traitements plus efficaces et plus spécifiques en fonction des facteurs de risque de mauvais pronostic: l’âge au moment du diagnostic de moins d'un an et de plus de neuf ans, des globules blancs de plus de 50 X 10^9^/L lors du diagnostic, une leucémie aiguë lymphoblastique à cellules T, une cytogénétique défavorable ( l'hypodiploïdie, la présence du chromosome de Philadelphie ou un réarrangement du gène mlL) et une maladie résiduelle minimale[14]. En revanche, le pronostic de LAM est toujours sombre à l'heure actuelle, mais la survie s'est toutefois significativement améliorée au cours des 30 dernières années en pédiatrie et pour le jeune adulte[15]. En effet, les taux de survie en pédiatrie sont passés de 3% au cours de la décennie 1970-1979 à 60% de nos jours [11, 15]. Les améliorations de la survie sont liées à une meilleure compréhension de la biologie moléculaire et cellulaire de la LAM, à l'intensification de la chimiothérapie d'induction de la rémission et des traitements de consolidation de même qu'au développement de meilleurs traitements de soutien, notamment en termes de transfusion et en matière de prévention des infections [15]. L'examen biologie utile et initial dans le diagnostique des leucémies quelque soit le type cellulaire se base toujours en premier lieu sur la cytologie qui est la clef du diagnostic dans le but de rechercher une infiltration de la moelle par des blastes. Des techniques spécifiques telles que la cytométrie en flux, la cytogénétique, la biologie moléculaire occupent une place cruciale dans la confirmation du diagnostique, mais aussi dans l'instauration du traitement et le suivi de la maladie résiduelle après traitement, mais sans pour autant pouvoir remplacer la cytologie dans la prise en charge de ces pathologies non seulement dans le processus du diagnostic mais aussi à cause de la non accessibilité de ces techniques spécifiques dans plusieurs zones particulièrement dans les pays en voie de développement [17]. Dans notre étude, l'examen cytologique du sang et de la moelle était concordant avec le résultat de la cytométrie en flux à 92,7% pour les LAL et 82,6% pour les LAM. Ces résultats sont comparable avec ceux trouvé dans une étude tunisienne [5]. L'immunophénotypage est indispensable pour confirmer le diagnostic des LA, rechercher une LA biphénotypique et éliminer une LAM indifférenciée (LAM0) [17]. Dans cette étude, le taux de concordance entre la cytologie et l'immunophénotypage était plus élevé pour les LAL que pour les LAM. Certaines études ont rapporté quela LAB évoque le plus souvent une LAM [5].

L’étude immunophénotypique permet la mise en évidence de divers antigènes de différenciation membranaire ou intra-cytoplasmique en confirmant l'appartenance à une lignée. En pratique, l'expression intracytoplasmique de CD79a ou de la chaîne epsilon de CD3 signe son engagement à la lignée lymphoïde, elle est associée à l'expression membranaire de la molécule CD19 pour la lignée B, Les autres antigènes associés qui apparaissent ensuite sont CD22, CD24 puis CD20. Concernant la lignée T, elles expriment en premier lieu la molécule d'adhésion CD7, dans une phase précoce pouvant conduire à un retour vers la lignée myéloïde. Les antigènes CD2 et CD5 apparaissent ensuite [17]. Dans notre étude, l'expression des CD 79a et CD19 était respectivement 87% et 81% des cas chez les patients diagnostiqués de LAL. La Co-expression des CD3 et CD79a a été observé également dans 9% des patients présentant la LAL-T. Chez les patients atteints de LAM une MPO positive avec expression de CD13 et/ou CD33 reste des critères d'appartenance vers la lignée myéloïde, la positivité de la MPO et celui du CD13 de nos patients était de 87% et 91% pour le CD33. Ces résultats sont concordants à ceux de la littérature [17].

## Conclusion

Le diagnostic des leucémies quelques soient le type se base toujours en premier lieu dans la pratique quotidienne sur la cytologie. L'immunophénotypage nous a permis de confirmer et/ou de redresser le cas difficile au niveau de la cytologie, mais aussi améliorer la prise en charge ainsi que le facteur pronostic. Le problème des LA dans le service d'hémato-oncologie pédiatrique reste un problème majeur. La prise en charge préventive et curative de ces patients nécessite les centres spécialisés.

## Etat des connaissance sur le sujet

- La leucémie est la plus fréquent des cancers pédiatriques et il existe deux principaux types de leucémie aiguë; la leucémie aiguë lymphoblastique et la leucémie aiguë myéloïde. La leucémie aiguë lymphoblastique est le cancer le plus fréquent chez les enfants et représente environ un quart de tous les cancers chez les sujets de moins de 15 ans tandisque les leucémies aiguës myéloïdes sont rares chez les enfants;
- Le diagnostic des leucémies aiguës repose avant tout sur des critères cytologiques et immunophénotypiques des blastes de la moelle osseuse. L'apport de l'immunophénotypage puis de la cytogénétique et enfin de la biologie moléculaire ont permet de décrire de plus en plus d'entités mais ces méthodes spécifiques sont couteuses et ne sont pas disponibles dans les centres hospitaliers des pays en voie de développement.

### Contribution de notre étude a la connaissance

- Cette étude montre les caractéristiques épidémiologiques, cliniques, hématologique, cytologiques et immunophénotypiques des leucémies chez les enfants au Maroc (Première étude qui s'intéresse sur ce sujet au Maroc);
- Cette étude montre un taux de concordance élevé entre les résultats de la cytologie à ceux de l'immunophénotypage. Donc la cytologie garde sa place dans le diagnostic des leucémies aiguës surtout dans les pays en voie de développement;
- Cette étude montre aussi l'importance de l'immunophénotypage dans le diagnostic des leucémies aiguës car cette technique nous a permis de faire une meilleure distinction entre les différents types leucémies aiguës.
